# Video-Assisted Thoracoscopic Surgery for the Stage II Pleural Empyema: A Prospective Observational Study

**DOI:** 10.7759/cureus.101952

**Published:** 2026-01-20

**Authors:** Akhileshwar Singh, Summaya Shikalgar, Sanjay Kolte, Jaisingh Shinde

**Affiliations:** 1 General Surgery, Sahyadri Super Speciality Hospital, Pune, IND

**Keywords:** hrct thorax, intercostal chest tube drain, pleural empyema, video-assisted thoracoscopic surgery (vats), video-assisted thoracoscopic surgical decortication

## Abstract

Background and objectives

Empyema thoracis is a condition in which purulent fluid collects in the pleural cavity. Video-assisted thoracoscopic surgery (VATS) is a minimally invasive technique that involves insertion of a thoracoscope through small incisions, or ports, in the chest wall. Despite modern diagnostic methods, pleural empyema remains a serious problem. The common causes are complications of pneumonia (fungal and mycobacterial), abdominal infections, or those following trauma and surgical procedures. The aim and objectives of this study are to evaluate the role and efficacy of VATS in stage II empyema in terms of duration of hospital stay, post-operative intercostal drain (ICD) removal, clinical and radiological recovery, and to evaluate the complications associated with the procedure.

Methods

This prospective observational study was carried out on a sample size of 31 patients fulfilling the inclusion and exclusion criteria, admitted to the General Surgery Department at Sahyadri Super Speciality Hospital, Pune, India, over a period of two years (January 2017 to December 2018). The patients were diagnosed with Stage II empyema as per a high-resolution computed tomography (HRCT) scan of the thorax. All patients underwent thoracoscopic decortication after the required pre-operative investigations. Pleural tissue and fluid were sent for biochemical, microbiological, and histopathological examination (HPE). All data were critically analysed to support the results and conclusions.

Results

The mean age of the cases was 52.5 years, with a male-to-female ratio of 1.58:1. Shortness of breath was the most common symptom (24 cases, 77.4%), followed by cough (20 cases, 64.5%). In the study group, diabetes was the most common comorbidity (11 cases, 35.5%), followed by hypertension (8 cases, 25.8%). Pre-operatively, the most common radiological finding was loculated pleural effusion (28 cases, 90.3%), followed by pleural thickening (20 cases, 64.5%) and split pleura sign (14 cases, 45.2%). Acute-on-chronic inflammation was the most common histopathological report of pleural tissue biopsy (16 cases, 51.6%). The majority of patients had an uneventful recovery (29 cases, 93.5%); however, there was a complication of pulmonary embolism (1 case, 3.2%) and elective intra-operative conversion to thoracotomy in 3.2% of cases. Stable patients were discharged with a posterior chest drain (16 cases, 51.6%), resulting in a reduced duration of hospital stay. The majority of patients were discharged within a week of surgery (19 cases, 61.3%) after confirming adequate lung expansion on chest radiograph.

Conclusions

Empyema thoracis should be suspected in patients with long-standing pneumonia or pneumonia unresponsive to antibiotic therapy. Ultrasound and HRCT scan are useful in diagnosis, whereas chest radiography is useful for monitoring postoperative lung re-expansion. Pre-emptive referral of patients for VATS will avoid multiple attempts at pigtail or chest drain insertion and prevent progression of the disease, thus reducing the hospital stay, morbidity, and mortality associated with the disease.

## Introduction

The term “empyema” is a Greek word meaning “pus-producing.” Empyema thoracis is a condition in which purulent fluid from infected tissue collects in the pleural cavity [1]. In the era of minimally invasive surgery, doing less is better for reducing patient morbidity. Thoracoscopy can be used for visualisation (pleuroscopy) or for performing surgical procedures. Surgical thoracoscopy is more commonly referred to as video-assisted thoracoscopic surgery (VATS). The first thoracoscopy was performed using a modified cystoscope by H.C. Jacobaeus [2]. Despite modern diagnostic methods and appropriate treatment, pleural empyema remains a serious problem. It most commonly occurs as a complication of pneumonia [3-5]. Other causes include complications of primary fungal or mycobacterial infections, or following abdominal infections, trauma [5], and surgical procedures.

Pleural empyema is classified into stage I (acute exudative phase, 0-7 days), stage II (transitional fibrinopurulent phase, 7-21 days), and stage III (chronic organising phase, >21 days), based on the chronicity of the disease process [6]. The main treatment modalities currently applied for pleural empyema are administration of antibiotics and drainage of infected pleural fluid. Despite numerous studies, the best management of pleural empyema is controversial, and the role of primary surgical intervention as a first-line approach is unclear.

The primary outcome of this study was to evaluate the role and efficacy of VATS in stage II empyema in terms of duration of hospital stay, postoperative intercostal drain (ICD) removal, and clinical and radiological recovery. The secondary outcomes included complications associated with the procedure, demographic factors, and etiological factors such as age, gender predisposition, laterality, symptoms, comorbidities, preoperative interventions, and histopathological and microbiological analyses. The study has been reported according to the STROBE guidelines.

## Materials and methods

Study criteria

This single-arm prospective observational study was conducted in the Department of General Surgery at Sahyadri Super Speciality Hospital, Pune, India, from January 2017 to December 2018. The Institutional Ethics Committee of Sahyadri Hospital issued approval for this study (approval no. ECR/493/Inst/MH/2013/RR-19). Consecutive patients were recruited from surgical admissions. The study included patients over 18 years of age, irrespective of gender, who were diagnosed with stage II empyema and underwent thoracoscopic decortication and drainage. Potential confounders included age, gender, comorbidities (e.g., diabetes), and preoperative interventions.

Exclusion criteria included patients in the late stage of empyema (stage III or >4 weeks from onset), inability to tolerate single-lung ventilation or poor PFTs (pulmonary function tests), recent history of myocardial infarction, coagulopathy, and empyema secondary to trauma. Individuals who were unwilling to undergo thoracoscopy were also excluded from the study. A total of 31 patients meeting the inclusion and exclusion criteria were enrolled. The study was approved by the Medical Research Committee at Sahyadri Super Speciality Hospital. All resources were institutional, and there was no external funding.

Methodology

In this prospective observational study, all patients with a history suggestive of empyema admitted to the Department of General Surgery underwent a thorough clinical evaluation, followed by routine diagnostic blood tests. Ultrasound and high-resolution computed tomography (HRCT) of the thorax were useful in confirming the diagnosis, whereas the patients' radiological progress was monitored by chest X-ray. Ultrasound was used to detect small effusions, loculations, septations, and details about fluid viscosity. HRCT was used to diagnose the stage of empyema: stage I, indicating lenticular fluid collection along with air space disease; stage II, suggesting pleural mass, atypical pleural fluid collections, pleural thickening, septations, and split pleura sign; and stage III, suggesting pleural peel with or without calcific pleuritis, lung entrapment, rib approximation leading to contraction of the affected hemithorax, and chest wall invasion. Patients were counselled, and informed consent was obtained prior to enrolling in the study. Patient-centric care was aimed for by using a multidisciplinary approach involving a pulmonologist, an anaesthetist, a physiotherapist, and the surgical team.

**Figure 1:**
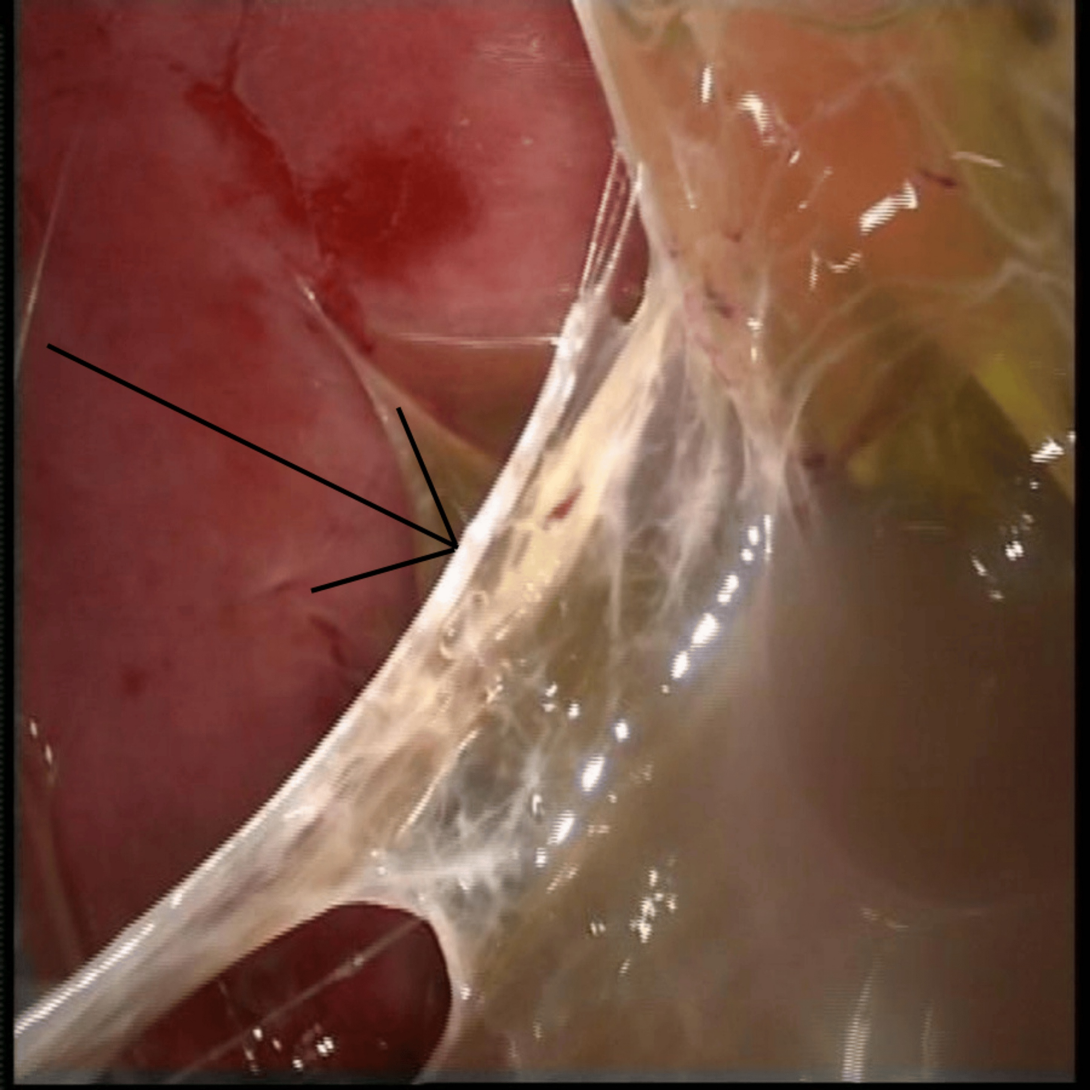
Fibrinous adhesions Intra-operative thoracoscopic view showing fibrinous adhesions within the pleural cavity (arrows indicate areas of dense adhesion).

**Figure 2:**
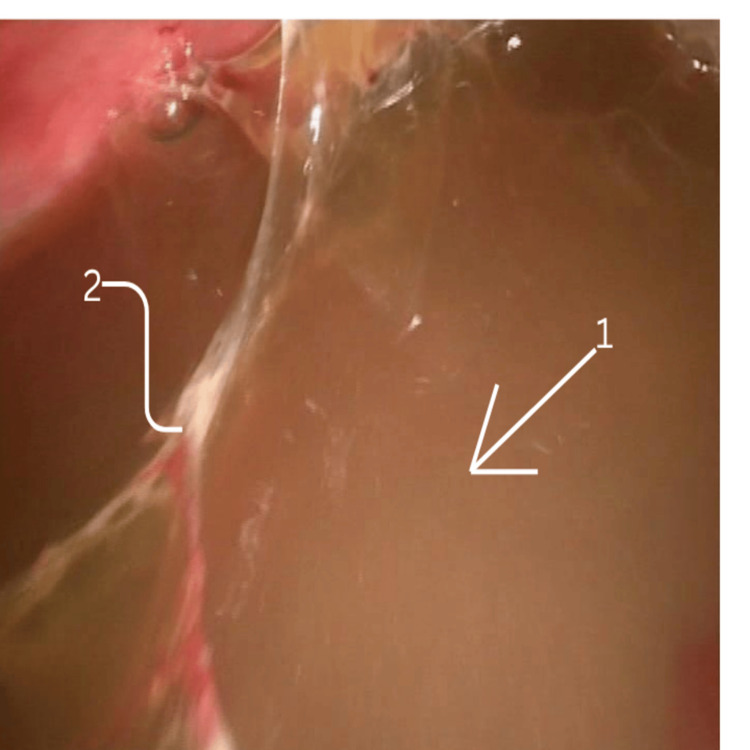
Pleural collection Intra-operative thoracoscopic image demonstrating multiple septations and loculated pockets of pus, and a large purulent pleural collection within the pleural cavity. 1) Arrow marks septations, characteristic of stage II fibrinopurulent empyema. 2) Thoracoscopic image demonstrating multiple septations and loculated pockets of pus within the pleural cavity, characteristic of stage II fibrinopurulent empyema.

**Figure 3:**
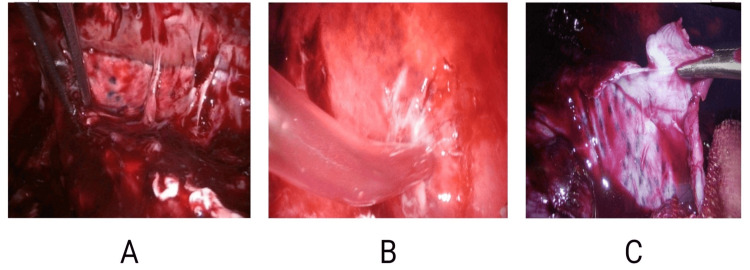
Key steps of video-assisted thoracoscopic decortication for stage II empyema A) Adhesiolysis using a sponge-holding forceps. B) Disruption of loculi and aspiration of pus with a suction catheter. C) Pleural decortication with Kelly forceps to release the visceral peel and achieve full lung re-expansion.

All patients were operated on under general anaesthesia, with single-lung ventilation achieved by a double-lumen endotracheal tube, in a standard right or left lateral position with a 45° tilt. The surgery was performed with the help of two to three surgical ports, with the first port placed in the triangle of safety and the remaining ports depending on the site of collection. Figure 1 demonstrates fibrinous adhesions within the pleural cavity, with digital markings added to highlight the areas of dense adhesion. Figure 2 shows the pleural fluid collection, with loculated pockets clearly indicated. Figure 3 provides a stepwise depiction of the decortication procedure, including adhesiolysis and removal of the fibrinopurulent peel, with markings used to identify the relevant operative steps.

The operations involved a combination of drainage, deloculation, and decortication. Adhesiolysis was done using a suction cannula to release flimsy adhesions, and Kelly forceps to release dense adhesions. Pleural tissue and fluid were sent for pathological, microbiological, and biochemical evaluation. Two ICDs were kept under vision, anterior and posterior to the lung.

A chest X-ray was done after the procedure, and repeated on the second postoperative day to look for lung expansion and residual collection. Spirometry was advised every two hours for better lung expansion. Patients were empirically started on broad-spectrum intravenous antibiotics (Amoxiclav), and the antibiotics were later changed as per culture reports. Follow-up continued until ICD removal and discharge (up to 21 days). To address potential sources of bias (e.g., selection bias), standardised protocols for assessment were used.

Statistical analysis

The sample size of 31 was determined in consultation with the institutional statistician using the effect sizes from the previously published study [7] and approved by the ethics committee.

The sample size was calculated with the help of the following formula:

$n = \frac{Z^2 \cdot p \cdot q}{(me)^2}$

where p = 0.11 (approximate incidence of postoperative complications), q = 0.89 (complement of p), Z = 1.96 (score at 95% confidence interval), and me = 0.11 (margin of error)

$n = \frac{(1.96)^2 \cdot 0.11 \cdot 0.89}{(0.11)^2} = 31.08 \approx 31$

Continuous variables were presented as mean ± standard deviation (SD), with minimum-maximum ranges. Categorical variables were presented as frequencies and percentages (n (%)). An exploratory Chi-square test was performed to examine the association between postoperative complications and duration of symptoms before presentation (≤14 days vs. >14 days). Groupings (e.g., age categories) were chosen for clinical relevance.

All statistical analyses were performed using IBM SPSS Statistics for Windows, Version 21.0 (Released 2012; IBM Corp., Armonk, NY, USA). All hypotheses were two-tailed, and a p-value of less than 0.05 was considered statistically significant. No adjustments were made for multiple comparisons, as this was primarily a descriptive observational study, with inferential tests applied only where relevant (e.g., association between complications and duration of presentation).

An exploratory logistic regression, adjusting for age and comorbidity (diabetes mellitus), was also performed to explore potential predictors of postoperative complications. Missing data (<5%) were addressed via listwise deletion; no loss to follow-up occurred, and no sensitivity analyses were performed due to the sample size. 

## Results

A total of 46 patients were assessed for suspected empyema thoracis. Of these, 40 were radiologically confirmed to have empyema. Nine patients were excluded due to stage III disease (n = 5), poor pulmonary function rendering them unfit for general anaesthesia (n = 2), or refusal of thoracoscopy (n = 2). Thirty-one patients were eligible, consented to VATS, underwent thoracoscopic decortication, and completed the follow-up period. All 31 patients were included in the final analysis (Table 1). Data completeness was verified before analysis. 

**Table 1:** Study patient selection flow All values are represented as ‘n’, indicating the number of patients. VATS, video-assisted thoracoscopic surgery

Stage	Description/Reason	Number of Patients
Patients assessed for empyema thoracis	Total patients screened	46
Diagnosed on imaging as empyema	Eligible based on radiological confirmation	40
Excluded	-	9
	Stage III empyema	5
	Poor pulmonary function/unfit for general anaesthesia	2
	Declined thoracoscopy	2
Eligible and consented for VATS	Agreed on surgical treatment	31
Underwent thoracoscopic decortication	Successfully completed planned procedure	31
Completed follow-up and included in analysis	Available outcome data	31

Among the 31 patients included in the study, the mean age was 52.5 ± 18.0 years, and the age range was 23-84 years. The study group consisted of 19 males (61.3%) and 12 females (38.7%), with a male-to-female ratio of 1.58:1. The mean duration of symptoms before presentation in the surgical outpatient department (OPD) was 13.2 ± 5.5 days, ranging from 3 to 24 days. The maximum number of patients (45.2%, n = 14) presented in the third week of illness (15-20 days). Shortness of breath was the most common symptom (77.4%, n = 24), followed by cough (64.5%, n = 20). Diabetes mellitus was the most common comorbidity, affecting 35.5% (n = 11) of patients, followed by hypertension in 25.8% (n = 8). Other comorbidities included asthma (6.5%, n = 2) and miscellaneous conditions (25.8%, n = 8; e.g., ischemic heart disease, chronic kidney disease, cerebrovascular accident). The right hemithorax was involved in 58.1% (n = 18) of patients. Preoperatively, the most common radiological finding was loculated pleural effusion (90.3%, n = 28), followed by pleural thickening (64.5%, n = 20) and the split pleura sign (45.2%, n = 14). Previous interventions were performed in 32.3% (n = 10) of patients, including pleural tapping (19.4%, n = 6), pigtail insertion (6.5%, n = 2), and ICD insertion (6.5%, n = 2) before presentation to the surgical OPD. Table 2 presents the various demographic and clinical parameters.

**Table 2:** Demographics and clinical parameters Values are presented as n (%) unless otherwise specified. IHD, ischemic heart disease; CKD, chronic kidney disease; CVA, cerebrovascular accident

Parameter	Value
Mean age (years)	52.5 ± 18
Age group (years)	
20–39	9 (29%)
40–59	10 (32.3%)
60–79	10 (32.3%)
≥80	2 (6.5%)
Gender	
Male	19 (61.3%)
Female	12 (38.7%)
Duration of symptoms before presentation (days)	
≤7	4 (12.9%)
7–14	12 (38.7%)
15–20	14 (45.2%)
21–30	1 (3.2%)
Presenting symptoms	
Shortness of breath	24 (77.4%)
Cough	20 (64.5%)
Comorbidities	
Diabetes mellitus	11 (35.5%)
Hypertension	8 (25.8%)
Asthma	2 (6.5%)
Others (IHD, CKD, CVA)	8 (25.8%)
Side affected	
Right	18 (58.1%)
Left	13 (41.9%)
Preoperative radiological findings	
Loculated pleural effusion	28 (90.3%)
Pleural thickening	20 (64.5%)
Split pleura sign	14 (45.2%)
Preoperative interventions	
Pleural tapping	6 (19.4%)
Pigtail insertion	2 (6.5%)
Intercostal drain insertion	2 (6.5%)
None	21 (67.7%)

Due to intraoperative difficulty, one patient underwent conversion to thoracotomy, and the remaining 30 patients were operated on by VATS. The most common histopathological examination (HPE) report was acute-on-chronic inflammation (51.6%, n = 16), and organisms could be isolated in 16.1% (n = 5) of patients. Postoperative complications occurred in 6.5% (n = 2) of patients, including pulmonary embolism in one patient (leading to delayed discharge at 18 days) and conversion to thoracotomy in one patient. The incidence of complications did not differ significantly across various groups of symptom duration before presentation (Chi-square test, p > 0.05). The anterior ICD was removed within four days in the majority of cases (74.2%, n = 23), with a mean removal time of 3.9 ± 1.2 days (range: 2-8 days). The posterior ICD was removed within nine days in 74.2% (n = 23) of patients, with a mean removal time of 7.7 ± 2.4 days (range: 4-13 days). The majority of patients (61.3%, n = 19) were discharged within one week of surgery, with a mean postoperative day of discharge of 6.8 ± 3.8 days (range: 2-21 days). Lung expansion improved by 87% ± 10% (95% CI: 83%-91%). Of the 31 patients, 51.6% (n = 16) were discharged with the posterior ICD in situ, and 48.4% (n = 15) were discharged after removal of both ICDs. Unadjusted results include 95% CIs, where applicable, and are summarised in Table 3. Given the single-arm design, no formal confounder-adjusted estimates were produced, though exploratory regression adjusting for age and comorbidity showed no material change. Categorical boundaries were defined as age <40, 40-59, ≥60 years; symptom duration ≤14 vs. >14 days; and empyema stage II vs. early III.

**Table 3:** Pleural biopsy and recovery All values are n (%) unless otherwise specified. ICD, intercostal drain

Parameter	Value
Histopathological examination (HPE)	
Acute-on-chronic inflammation	16 (51.6%)
Acute-on-chronic necrotising inflammation	7 (22.6%)
Tuberculosis (caseating granulomas)	5 (16.1%)
Others	3 (9.7%)
Postoperative complications	
None	29 (93.5%)
Pulmonary embolism	1 (3.2%)
Conversion to thoracotomy	1 (3.2%)
Anterior ICD removal (days)	Mean: 3.9 ± 1.2 (range: 2–8)
2–4	23 (74.2%)
5–8	8 (25.8%)
Posterior ICD removal (days)	Mean: 7.7 ± 2.4 (range: 4–13)
4–6	12 (38.7%)
7–9	11 (35.5%)
10–13	8 (25.8%)
Postoperative day of discharge	Mean: 6.8 ± 3.8 (range: 2–21)
2–6	19 (61.3%)
7–14	11 (35.5%)
15–21	1 (3.2%)
Discharge with posterior ICD in situ	16 (51.6%)

The incidence of postoperative complications did not differ significantly across groups of symptom duration before presentation (Chi-square = 0.42, df = 1, p = 0.52). Exploratory logistic regression, adjusting for age and comorbidity, showed no statistically significant predictors of postoperative complications (age: OR 1.12, 95% CI: 0.21-5.89, p = 0.89; diabetes: OR 1.35, 95% CI: 0.24-7.63, p = 0.73). Table 4 presents an exploratory logistic regression for predictors of postoperative complications.

**Table 4:** Exploratory logistic regression for predictors of postoperative complications An exploratory logistic regression examined the relationship between postoperative complications and clinical factors (age and diabetes mellitus). Given the small number of events (n = 2), statistical estimates are unstable and should be used for descriptive purposes only. OR, odds ratio; CI, confidence interval

Variable	Odds Ratio (OR)	95% CI	p-value
Age ≥ 60 years	1.12	0.21–5.89	0.89
Diabetes mellitus	1.35	0.24–7.63	0.73

## Discussion

This single-arm, unbiased observational study analysed 31 cases of empyema selected according to defined inclusion and exclusion criteria. Shortness of breath was observed as the most common symptom. Dyspnoea in empyema is attributed to several factors, including impaired gas exchange, disrupted respiratory mechanics, and elevated intrapleural pressure [8]. VATS allows direct visualisation of the pleural cavity, enabling decortication and HPE. The management’s end target is to achieve early rehabilitation through re-expansion of the trapped lung.

We observed right-sided involvement in 18 (58.1%) cases. Similar results were found by Banga et al. [9] from AIIMS, New Delhi. This may be attributed to the shorter and straighter course of the right bronchus. One patient in our study had bilateral involvement.

Similar studies by Banga et al. [9] from AIIMS, New Delhi, and Lingayat and Wankhade [10] from GMC, Aurangabad, have shown male predominance, supporting this study. Males are more prone due to mechanical stresses, strenuous work, smoking, and chronic obstructive pulmonary disease (COPD).

In the study, the majority of the patients (61.3%, 18) were discharged within six days, with post-procedure hospital stay ranging from 2 to 21 days. This was achieved through optimal intraoperative clearance and intensive postoperative care, including spirometry and physiotherapy. The average time of anterior ICD removal was four days, and posterior ICD removal was eight days. A similar study by Lawrence et al. in the UK [11] showed an average hospital stay of five days and an average time for ICD removal of four days. During this study, the hospital stay was reduced by discharging stable patients with posterior ICD in situ (51.6%) in view of persistent drain output, followed by removal during follow-up on an OPD basis. In a similar study by Landreneau et al. [12], 12% of patients were discharged with ICD in situ.

Microbiological analysis isolated organisms in five (16.1%) cases, but no predominant organism could be identified. Most patients had physician visits or treatment with antibiotics, resulting in negative culture reports on pleural fluid or tissue analysis. Similar studies conducted by Kundu et al. [13] from Kolkata and Banga et al. [9] from Delhi showed isolation of organisms in 42% of cases, with *Staphylococcus aureus* being the most common (23%), followed by Gram-negative bacilli. Diabetes was the most common comorbidity, involving 11 (35.5%) cases. A similar study by Kundu et al. [13] had 28.3% diabetic patients.

Raised WBC count was observed in 77.4% of patients, with an average count of 15,061 during the first hospital visit. Similar results were observed by Kundu et al. [13], with a raised average WBC count of 14,877 during the first presentation. We observed a 3.2% conversion rate to thoracotomy due to intraoperative difficulty. Similar studies by Angelillo Mackinlay et al. [14] and Luh et al. [15] have shown a 10.3% conversion rate. The common reasons for conversion were bleeding, adhesions, and disease progression. Earlier studies have suggested that early intervention by thoracoscopy is beneficial and that the conversion rates are higher as the disease progresses [16].

The findings are primarily generalisable to adults with stage II empyema managed in centres with established thoracoscopic expertise. As patients with advanced disease or significant comorbidities were excluded, outcomes may overestimate effectiveness in broader or more complex populations. The single-centre design and short-term follow-up also limit applicability to other settings and long-term trajectories. This study can be applied in similar clinical environments where timely VATS decortication is feasible.

Limitations

This study has limitations, including a small sample size, absence of a comparison arm, and short follow-up duration, which may introduce imprecision and potential bias. Selection bias may have favoured patients fit for surgery, likely overestimating outcomes to a moderate extent. A short follow-up may have missed late complications, biasing results towards greater improvement. Potential confounding could not be fully controlled due to the single-arm design, and its direction is variable, depending on the factor.

No formal correlational or comparative analyses beyond the Chi-square test were performed due to the descriptive, single-arm nature of the study and small sample size. Future larger studies could explore additional associations. The small sample size limits the power to detect statistically significant associations. These limitations should be considered when interpreting the results and applying them to broader populations.

## Conclusions

Empyema thoracis should be suspected in patients with long-standing pneumonia or pneumonia unresponsive to antibiotic therapy. Ultrasound and HRCT scans are useful in diagnosis. A chest X-ray is useful for monitoring postoperative radiological progress. Pre-emptive referral of patients for VATS will avoid multiple attempts at pigtail or chest drain insertion and prevent progression of the disease. This will thus reduce the hospital stay, morbidity, and mortality associated with the disease.

In this study, VATS decortication was associated with favourable clinical outcomes in patients with loculated, complex, fibrinopurulent parapneumonic effusions, including good lung re-expansion and acceptable complication rates. Hence, VATS is useful in treating stage II empyema. While previous comparative studies have suggested that VATS may provide outcomes comparable to thoracotomy, with advantages such as less postoperative pain and faster recovery, our study was not designed to evaluate comparative effectiveness. Delayed intervention is associated with increased conversion to open surgery, more procedures, more radiographs, more cost, and an increased length of stay.
